# Use of paraffin oils in agriculture and beyond: back to the future

**DOI:** 10.1007/s11356-022-24059-5

**Published:** 2022-11-18

**Authors:** Georgia V. Baliota, Christos G. Athanassiou

**Affiliations:** Laboratory of Entomology and Agricultural Zoology, Department of Agriculture, Crop Production and Rural Environment, University of Thessaly, Phytokou Str, 38446 Volos, Magnesia Greece

**Keywords:** Mineral oils, Insecticides, Pest control, Integrated pest management, Arboriculture

## Abstract

Paraffin (petroleum) oils have been used for many years for their insecticidal properties, but relatively little research had been conducted towards their introduction into the agricultural praxis, due to their potential phytotoxic effects. In the recent years, however, there has been an increased interest in petroleum-based pesticides due to their compatibility with integrated pest management (IPM) programs. Various improvements in the refinement methods have enhanced the manufacture of commercial products with many advantageous features over the original oil formulas. However, literature is still lacking of a general overview about the applicability of newly introduced commercial petroleum oils in agriculture and their compatibility with modern pest management practices. Therefore, the present work aims to depict the current status of petroleum oils in arboriculture and beyond, providing an in-depth analysis of their insecticidal properties with respect to the knowledge gained over the years about the factors responsible for the pesticidal efficacy and the phytotoxic activity of petroleum-derived oil insecticides. Moreover, commercial aspects of petroleum oil formulations and their toxicological profile to non-target organisms have also been addressed through the current legislation in EU and the USA.

## Introduction

Petroleum oils were known as effective means of controlling certain insect pests as early as 1890 (Riley 1891, 1892), and their applications were rapidly adopted in agriculture during the late nineteenth century, probably because of the oils’ general availability. The first petroleum-based insecticides referred to crude and undiluted oil emulsions and were proposed to farmers for applications upon dormant trees, since phytotoxicity issues to the foliage and fruits were frequently observed (Volck 1903; Gray and de Ong 1926; English 1928). Few years later, specific refining methods (Hurst 1925; Gurwitsh 1927; Gruse 1928) improved further the chemical and physical properties of petroleum oils. Various oil grades were produced afterwards, and classified based upon the major residue left after distillation, as paraffin, asphaltic (or naphthene), or mixed (if containing both asphalt and paraffin base) (Swingle and Snapp 1931).

Such oils gained prompt popularity due to their increased safety to plant tissues coupled with their high insecticidal value (de Ong et al. 1927; Volck 1929). Thus, extensive work with over 50 published researches within a decade, evaluating the effectiveness of oil emulsions in applications mostly upon fruit trees to control scale insects, aphids, psyllids, leaf rollers, and mites. However, phytotoxic effects including leaf scorching and browning, defoliation, reduced flowering and stunted growth continue to be an issue of major importance (Rohrbaugh 1941; Wedding et al. 1952). Detailed descriptions about the preparation of the applicable oil formulas or their utilization in arboriculture during these years can be found in the work of de Ong et al. (1927) and Swingle and Snapp (1931). Studies of this period were considered as foundation for further research towards the use of petroleum based insecticides in agriculture (Agnello 2002), although there was only a basic understanding about the chemistry of petroleum oils.

The greatest improvements towards the understanding of the efficacy and chemistry of petroleum oils occurred in the first half of the twentieth century. During this period, both oil-producing companies and applied entomologists tried to specify the responsible components for the phytotoxicity and the insecticidal efficacy, aiming to develop oil formulations for commercial use, particularly on fruit trees. Thus, the subsequent research focused mainly in the assessment of the phytotoxic effects of petroleum oil products, while their insecticidal efficacy was rather of secondary importance. Thanks to this extensive research, phytotoxic responses from both macroscopic and microscopic perceptive were known by 1970 (Baker 1970). The interaction between the constituents and amount of the oil, the environmental conditions and the plant tissues involved were described in details in the same review. Nevertheless, research was scarce in the next two decades. Surprisingly, it was not later than 1990 when petroleum oils were registered for the first time as active ingredients for pesticide use by the US Environmental Protection Agency (US EPA). Over one hundred similar pesticides were registered afterwards (USDA 2015).

In recent years, the necessity to place insect control under the principles of integrated pest management (IMP) reinvigorate the interest of research about the petroleum-based pesticides. Modern technology allow us to blend, purify, synthesize and thus, produce customized petroleum derived oils. Exploiting these technological advantages in conjunction with the current knowledge about their pesticide performance led to the creation of commercial formulations able to satisfy a specific range of physical and toxicological properties, which are also considered as advantages over the use of conventional pesticides.

Hence, newly introduced products derived from crude petroleum oils are among the most researched formulations in the last two decades, especially in the field of ecofriendly management approaches. The objective of the recent research is to broaden the petroleum oils’ spectrum of efficacy, evaluating their impact from both insecticidal and phytotoxicological perceptive against principal pests of a wide range of herbaceous species, including annual vegetables and fruits, cereals, ornamental plants, and others. Nevertheless, petroleum oils have been established as permanent components of pest management strategies only in arboriculture, with research to be focused mainly towards this direction.

## Chemistry of petroleum oils

### Chemical composition and refining process

Crude petroleum oils are complex mixtures of hydrocarbons, referred mostly to paraffinic (linear and branched alkenes), naphthenic (alkyl-substituted cyclo-alkanes) or aromatic (including polynuclear aromatic (PAHs)) hydrocarbons. Minor amounts of other compounds may also be included, such as sulfur, nitrogen, oxygen- and/or metal-containing compounds. Given the biological process in which the crude oils are formed, i.e. organic matter broken down under high pressure and temperature, the chemical composition of petroleum oils vary considerably among geographical regions, even among wells within the same area (Agnello 2002). Due to their complexity, it is not feasible to resolve such mixtures into individual components, but only to quantify the concentration of certain compounds, using gas chromatography methods (EFSA 2012). Nevertheless, since modern technology can offer standard processing specifications, the composition of a commercial formulations is linked mostly to the refinery history of the crude oil rather than its origin.

In order to convert a crude petroleum oil into a useful compound, catalytic and thermal methods are applied, resulting in thousands of chemical reactions during the refining (Neumann and Rahimian 1984). The derived petroleum distillates contain now a wide variety of hydrocarbons and other substances, thus requiring further refining to remove the phytotoxic compounds and other impurities, while keeping in the compounds with the desirable action (USDA 2019). Details of the most prevalent processes used for distillation and refinery of crude petroleum oils are provided in the Technical Evaluation Report of Mineral Oil by the USDA (2015).

Since highly specialized chemical processing equipment and methodology is required to produce the desired oil refinery, the National Organic Standards Board (NOSB) determined in 1995 that petroleum oils are synthetic substances with distinct chemical and physical patterns. Thus, specific protocols have been established by the American Society for Testing Materials (ASTM), to promote and standardize values and methods to characterize petroleum oils with regard to their suitability for pesticide utilization (Agnello 2002; USDA 2015). Among the most common of the properties are the boiling point, the boiling range, density, vapor pressure, molecular weight, viscosity, and unsulfonated residues (UR). The methods used to determine the majority of these values are well described by Agnello (2002).

The UR value indicate the percentage of the stable and non-phytotoxic saturated hydrocarbons in the oil. Contrariwise, the remaining quota corresponds to unsaturated hydrocarbons and other reactive impurities that remained after the distillations and refining processes and which are directly related to the toxicological effects the oils have on plant tissues. Thus, the UR value is a good measure of the degree of refinement, given that highly refined oils have UR above 99%, and indicates the phytotoxic potentials of the oil, although does not provide additional information about its pesticide efficacy (Agnello 2002; Borgan et al. 2006).

Viscosity in the petroleum industry is expressed through the time required for a given volume of oil to flow via a standard opening under specific temperature and pressure conditions. In terms of pest management, viscosity of sprayable oils tend to have a range from 60–100 centistokes (at 37.8 °C) (Davidson et al. 1991), and the ones with higher viscosity are expected to have greater pesticidal efficacy. Nevertheless, viscosity alone is not an absolute pesticide indicator, since the flow rate can be affected by both molecular size and shape, values that can be reflected through oils’ molecular weight.

The molecular weight of petroleum oils is expressed through the median equivalent n-paraffin carbon number (nCy), and it has been widely adopted recently in the market (Stadler and Buteler 2009). This specification is defined by nCy, where “n” denotes a normal paraffin (alkane) molecule and “y” denotes the number of carbon atoms (Kuhlmann and Jacques 2002). Thus, the specification indicates the equivalence in distillation temperature between the commercial oil formulation and the boiling point of a normal (n) straight-chain paraffin with “y” carbon atoms (Agnello 2002). In general, lighter oils have lower carbon number, i.e. an nC21 oil is lighter than an nC24 oil. The customary carbon number values of spray oils are nC21, nC23, nC24, and nC25, range in which spray oils combine low phytotoxicity with insecticidal effectiveness.

The boiling or distillation range is another method to determine the purity of a given petroleum oil, by boiling of a sample under controlled heating and atmospheric pressure. The range (in degrees Fahrenheit) is limited between the boiling point of the oil components at 10 and 90 percent distillation. A narrow boiling range is evidence of a more uniform composition, thus the rate of pesticidal efficacy and safety to plants is more standardized, compared with oils exhibit a wider boiling range. In addition, the boiling point is also an indicator about the oil volatility from the plant surface and insect’s body, hence may be a factor of importance in the pesticide efficacy and the phytotoxicity. Oils with lower boiling points volatilize more quickly after the application and thus no significant interaction occurs with plant tissues, in comparison with oils of higher boiling points. Nevertheless, there is a specific range of volatility an oil must exhibit (412–468 °F) (Borgan et al. 2006), since higher or lower volatility levels lead to evaporation before the oil penetrate into the pest’s body or to adhere on the cuticle without any insecticidal effect, respectively.

### Mode of action

The first reports about the components responsible for the insecticidal effect of petroleum oils pointed out the different mode of action of saturated and unsaturated hydrocarbons. It was believed that saturated hydrocarbons of oils demonstrate a physical action by blocking the spiracles and thus causing suffocation, whereas unsaturated hydrocarbons have toxic effects in insect and plants arbitrarily, by penetrating and corroding their tissues (Gray and de Ong 1926; de Ong et al. 1927; Shepard 1939; Najar-Rodríguez et al. 2008). Additionally, the majority of the early-stage petroleum oils also contained volatile compounds, and acted as fumigants against pests (Moore and Graham 1918). As such, only the saturated hydrocarbons were considered as profitable components and their quota has been associated with the insecticidal value of petroleum oils ever since. A wide historical perceptive of the insect toxicology of petroleum oils depending on their physical characteristics can be found in the review of Taverner (2002).

The mode of entry and the mechanisms under which petroleum oils act as insecticides and ovicides are yet a subject of debate, despite the improvements achieved in oil technology. In an attempt to link the functional role played between the insect physiology and oil physics, Taverner (2002), Stadler and Buteler (2009) and Buteler and Stadler (2011) addressed the known factors that govern the routes of entry and modes of action of oils in the insect body, via reviewing the literature. The same authors have compared and discussed adequate data and methodologies, concluding to suggestions about the most likely modes of action of the proposed theories throughout the years. Surprisingly, the most prominent theory of suffocation, i.e., interference of oil in the normal gaseous exchange of the pest’s respiration (de Ong et al. 1927; Davidson et al. 1991; Najar-Rodríguez et al. 2008), may be the key factor only under particular conditions, relating to oils and target pest species. Hence, it has been postulated that the mode of action of petroleum oils is more complicated that it was initially believed.

Observations of the symptomatology caused by oil toxicity suggest that a range of responses incurred in insect’s body after the exposure. Treatments with petroleum oils may cause knockdown due to suffocation, alter the color, the permeability and the dehydration of the cuticle, discourage feeding and egg deposition, disrupt the nervous system, or affect the abdomen contractions of the insects (Mazzella et al. 2005; Najar-Rodríguez et al. 2008; Buteler and Stadler 2011). The insecticidal activity of low polarity substances, such as petroleum oils, starts after their penetration via the insect cuticle, waxes or pore canals (Gerolt 1983). In contrast to conventional insecticides, the action mechanisms of petroleum oils appear to not bind to specific receptors (Buteler and Stadler 2011; Damavandian 2016). Hence, toxicity of oils depends on surface phenomena, biochemical or metabolism traits and on taxa and stage of the target species. Nevertheless, the synthesis, chemical purification and refining as well as the application rates and methods of oils are also insecticidal factors.

Penetration through the spiracle-tracheal (respiratory) system is one of the most accepted theories on the mode of action of petroleum oils, but only when adults or larvae are over-sprayed or dipped in oil (Stadler et al. 1996; Taverner et al. 2001; Sugiura et al. 2008). The inflow of oils into the air passages (tracheae) appears to induce mortality of insects due to asphyxia (by occlusion of tracheae). This physical mode of action is depended on the physical properties of oils such as their viscosity and nCy, and the dimensions of insect tracheae (Najar-Rodríguez et al. 2008; Buteler and Stadler 2011).

Petroleum oils penetrate the insect body most likely through the insect cuticle (Najar-Rodríguez et al. 2008; Buteler and Stadler 2011). The structural and chemical similarities between the saturated hydrocarbons of petroleum oils and the cuticular lipids result in interactions between the two substances (Wigglesworth 1941; Najar-Rodríguez et al. 2008). When in contact, the saturated hydrocarbons dissolve the lipids or move through the intercellular paces in the cuticle. Hence, the symptoms observed referred to changes in the texture (softening) (Stansly et al. 2002) and the melting point of the cuticular wax layer (Hagg 1969; Rourke and Gibbs 1999), or even in completely cuticular dewaxing (Lockey 1988). Following the unequal wax distribution, penetration of variant apolar compounds may be allowed, referred not only to petroleum oils but also to other synthetic insecticides. At the same time, mortality due to dehydration may be observed, as waxes originally regulate the water loss of insects body (Ebeling 1974; Lockey 1988; Hadley 1994), with oils having higher molecular weight to require longer period for desiccation (Taverner 2002). The degree of penetration depends primarily on the physical structure of the cuticular surface (Halley 1994). However, external conditions may also be a factor, since the miscibility of petroleum oils and cuticle waxes can be affected by temperature (Rowlinson and Freeman 1961). Afterwards, the type and concentration of oil, the insect species, and their developmental stage are the main key factors for further spread of oils with toxic effects inside the body of the insect (Buteler and Stadler 2011). The literature about the mode of entry of petroleum oils has been recently reviewed and details towards this direction can be found in the work of Buteler and Stadler (2011).

Once petroleum oils penetrate the cuticle or the tracheal system, apparently diffuses and accumulate within lipid-containing tissues (mostly in fat bodies), the hind gut and the central nervous system. Najar-Rodríguez et al. (2008) suggested that the accumulation of oils into the fat bodies may affect the energy supply provided by the tissues and eventually leads to insect death. However, the authors did not tested this theory. When oil concentrates within the nerve ganglia, insects may display symptoms of loss of coordination of motor activities and/or a dramatic reduction in synaptic transmission inside the central nervous system (Taverner et al. 2001). The nerve disruption may be due to the displacement of protective neural lipids affecting the nerve activity by increasing membrane permeability to ion exchange (Taverner et al. 2001). More details about the cellular disruptions via treatments with petroleum oils can be found in the work of Najar-Rodríguez et al. (2008).

Antifeedant properties of petroleum oils and starvation have also been documented by several authors (Baxendale and Johnson 1988; Beattie et al. 1995; Najar-Rodríguez et al. 2007). Additionally, reduction in oviposition has been observed for a variety of pest species and plants (Riedl et al. 1995; Rae et al. 1996; Weissling et al. 1997; Fernandez et al. 2001; Mensah et al. 2001, 2002). The residual film of petroleum oil upon the surfaces may have a repellent effect in insect, preventing them from attaching to plants for feeding or oviposition (Trammel 1965). Liu et al. (2001) addressed the influence of molecular weight and nCy value has in the persistence of oil molecules on sprayed surfaces, since petroleum oils with high molecular weight demonstrate better and longer activity as deterrents.

Even when petroleum oils may not successfully inhibit insects to oviposit, they can be used as ovicides. Smith (1952) was among the first who summarized the theories regarding the activity of oils in the eggs. He proposed that the oils interfere mechanically with the normal exchange of gases and water between the egg and the external environment, interfere with enzyme or hormone activity inside the egg, prevent hatching by hardening or softening and dissolving the outer covering, or penetrating the egg causing coagulation of the protoplasm. It was also pointed out that the less reactive saturated hydrocarbons had greater ovicidal activity than that of the unsaturated hydrocarbons (Buteler and Stadler 2011). Petroleum oils with molecular weight > 320 (C23) (which volatilize little over a 24-h period) are considered the most effective ovicides (Pearce and Chapman, 1952; Fiori et al. 1963; Taverner 2002).

## Application scenarios and target species

Lately, petroleum oils have gain an important role in the control of pests worldwide. In the course of the technological development, the evolution of the refining pathways have broaden not only the pesticidal efficacy but also the range of hosts in which petroleum oils can be applied. Since they are considered as natural pesticides, they have been associated with integrated pest management (IPM) approaches (Beattie and Smith 1997; Mensah et al. 2004). This is a very advantageous feature, in the light of the scarcity of –reduced risk pesticides nowadays. Furthermore, with respect to their physical mode of action, petroleum oils are frequently mentioned in resistance management schemes, since they are part of a group to which resistance has not been reported yet (Willett and Westigard 1988; Borgan et al. 2006).

### Use in orchards

Commercial petroleum oils are generally blended using emulsifier (mixing agent) with water to formulate aqueous spray formulations for easier applications (Helmy et al. 2012). Such formulations have been found to provide the same insecticidal efficacy compared with a conventional broad spectrum of insecticides (Liang et al. 2010; Leong et al. 2012; Damavandian and Moosavi 2014). In field trials, 5-year-old citrus trees infested by the citrus leafminer, *Phyllocnistis citrella* Stainton (Lepidoptera: Gracillaridae) were sprayed with commercial petroleum oils and imidacloprid, chlorpyrifos and abamectin in concentrations ranged from 100 to 900 ml of insecticide in 100 l of water (0.1–0.9% v/v) (Damavandian and Moosavi 2014). The results shown that a considerable damage reduction was achieved regardless of the insecticide used, but the results were comparable only when the oils applied at a rate of ≥ 0.65% v/v in water (Damavandian and Moosavi 2014). It was also stated that the cost of using petroleum oils was the least compared to the conventional insecticides. The red scale, *Aonidiella aurantii* (Maskell) and the purple scale, *Lepidosaphes beckii* (Newman) (Hemiptera: Diaspididae), two armored scale pests of citrus were effectively controlled after applications with an nC24 petroleum oil, carbaryl or methidathion, with lethal effects to be similar among the three insecticides (Liang et al. 2010). One application of 1% v/v petroleum oil for the control of the first generation nymphs of the San Jose scale, *Diaspidiotus perniciosus* (Comstock) (Hemiptera: Diaspididae), in almond and apple orchards in spring reduced the number of nymphs at a level similar to that of 0.07% v/v pyriproxyfen, 0.05% v/v phenoxycarb, or 0.08% v/v chlorpyrifos, but only at low level infestations (Sazo et al. 2008).

Intriguingly, the comparison of two pest management types, i.e., applying just petroleum oils vs. usage of synthetic pesticides on the activity of *P. citrella* in mature citrus orchards, indicated that there is more *P. citrella* damage in orchards under presence of synthetic pesticides than in orchards in which the synthetic pesticides were not used for years (Damavandian and Moosavi 2014). In another study, although applications of petroleum oils alone could not be considered as economically sustainable program in plum orchards, the addition of a low number of spinosad applications in the schedule provided good protection of fruits against the plum moth, *Grapholitha funebrana* Treitschke (Lepidoptera: Tortricidae) (Rizzo et al. 2012). Moreover, since applications of spinosad were fewer, the harmful side effects on beneficial arthropods, such as bees and natural enemies, were proportionally reduced. Similar results were obtained by the study of Taverner et al. (2011) against the light brown apple moth, *Epiphyas postvittana* (Walker) (Lepidoptera: Tortricidae). On the other hand, the combination of petroleum oils with spinosyn A and D, diacylhydrazin or pepper and garlic extracts increases only slightly the mortality of *P. citrella* (Amiri-Besheli 2009). Several studies are in support of the transition of synthetic pesticides to petroleum oils on the orchards since, apart from the effective control achieved, petroleum oils do not lead to emergence of new pest strains and simultaneously preserve the natural enemies (Chueca et al. 2010; Liang et al. 2010; Leong et al. 2012; Damavandian 2016; Sepasi et al. 2018). Similarly, there were no differences in the efficacy of a narrow-ranged petroleum oil (0.85% v/v) and hexythiazox (0.075% v/v plus 0.5% v/v oil) to suppress a natural outbreak of the citrus red mite, *Panonychus citri* (McGregor) (Acari: Tetranychidae) on 10-year-old sweet orange trees (Damavandian 2007). With respect to the reports about the feasible resistance of *P. citri* to several acaricides, including hexythiazox (French and Hutchinson 1980; Yamamoto et al. 1995, 1996), the successful pest control supports the use of petroleum oils in resistance management strategies.

A 3-year pest management program on apple orchards based entirely on treatments with a commercial paraffinic oil (nC23, UR 92%) proved overall successful by providing some additional control against the codling moth, *Cydia pomonella* (L.) (Lepidoptera: Tortricidae), and greatly suppressed certain secondary pests, such as the leafroller, *Pandemis pyrusana* Kearfott (Lepidoptera: Tortricidae), the white apple leafhopper, *Typhlocyba pomaria* McAtee (Hemiptera: Cicadellidae), the woolly apple aphid, *Eriosoma lanigerum* Hausman (Hemiptera: Aphididae), and some phytophagous tetranychid and eriophyid mites (Fernandez et al. 2005). In a following study, Fernandez et al. (2006) reported that when the same treatments targeted the apple powdery mildew, *Podosphaera leucotricha* (All. and Evherh.) Salm. (Erysiphales: Erysiphaceae), the mullein bug, *Campylomma verbasci* (Meyer-Dur) (Hemiptera: Miridae) and the rosy apple aphid, *Dysaphis plantaginea* (Passerini) (Hemiptera: Aphididae), adequate suppression was achieved only in one out of the three years of the study. Nevertheless, nymphs of *T. pomaria*, the apple rust mite, *Aculus schlechtendali* (Nalepa) (Acarida: Eriophyidae), the western predatory mite, *Galandromus occidentalis* (Nesbitt) and tetranychid mite populations were consistently suppressed. These findings are in accordance with the laboratory trials of Damavandian (2007), who collected *P. citri* from infested citrus orchards and treated them with concentrations ranged from 0.1 to 1% v/v of a narrow-ranged petroleum oil (nC23, UR 94%) diluted in water. In that study, the author reported that even the lowest concentration caused 65% mortality on mites after 24 h of exposure, whereas the control had only 9% mortality (Damavandian 2007). Additional experimentations on 2-year-old sweet orange trees that were infected artificially with *P. citri* shown that the mixture of 0.85% v/v of the same oil in water was sufficient to reduce the pest without phytotoxic risks (Damavandian 2007).

The stage-specific mortality effects of petroleum spray oils in the arrowhead scale, *Unaspis yanonensis* (Kuwana) (Hemiptera: Diaspididae) and the pink citrus rust mite, *Aculops pelekassi* (Keifer) (Acari: Eriophyoidea) were evaluated by Kim et al. (2010), who observed significantly different mortality rates depending on the stage of the insects at a laboratory scale. Moreover, there were indications of abnormal morphology and loose attachment of the scale cover of *U. yanonensis* caused by petroleum oil, but additional work was required to confirm this observation. Satisfactory control was also achieved against adults and nymphs of the scales *Parasaissetia nigra* (Nietner) and *Pulvinaria floccifera* (Westwood) (Hemiptera: Coccidae), when the commercial Super Misrona oil 95% EC (95% paraffinic oil w/w, UR 92%.) was applied in guava and mango trees at a rate of 1.5% v/v., for two years in the row (Abd-Rabou et al. 2012). The black scale, *Saissetia oleae* (Olivier) (Hemiptera: Coccidae) on olive trees and *P. citrella* on lemon trees, were effectively controlled by petroleum oils, as reported by Nicetic et al. (2011). Adequate control of eggs, protonymphs and adults of the two-spotted spider mite, *Tetranychus urticae* Koch (Acari: Tetranychidae) was achieved with spray applications of commercial petroleum oils between 15 μg oil/cm^2^ and 30 μg oil/cm^2^ under laboratory trials (Chueca et al. 2010).

Chueca et al. (2010) compared the insecticidal efficacy of commercial petroleum oils with different molecular structures at variant consentrations and found that there was a corresponding increase between the concentration, the n-paraffin carbon number, the coverage, the mean area of impacts and the insecticidal efficacy of the tested oils. Nicetic et al. (2011) attributed the low insecticidal performance of a plant-derived oil (canola oil) with nC55 to the different structure of molecules, as compared with two paraffinic oils (nC24). The authors stated that the very large size of molecules of canola oil resulted in very high viscosity, high distillation temperature, low volatility, and thus to lower penetration into the respiratory tract of the pest tested. On the other hand, Liu et al. (2001) reported that the nC25 petroleum oil was more effective than nC17 and nC22 oils on the oviposition by *P. citrella*. In addition, the same authors reported that the aromaticity and emulsifier concentration of the oils had no effects as oviposition deterrents. Similar results were obtained by Rae et al. (2000) in 2-year spray applications upon commercial sweet orange and pummelo orchards, since the nC27 petroleum oil appeared to be the most effective of the nC23 or nC27 oils against mites and scales. No phytotoxicity of the sprays was reported, in terms of leaf and fruit drop, fruit yield and the external quality of the fruit.

Complete oviposition deterrence for up to 3 weeks was observed in pear psylla, *Cacopsylla pyri* (L.) (Hemiptera: Psyllidae) when a 9-year-old pear orchard was sprayed with an aqueous mixture containing 1% v/v of a commercial summer oil (Erler 2004). Observations by the authors suggested that adult *C. pyri* had ambulatory difficulties on oil surfaces. Other studies are in conformity with these results (Zwick and Westigard 1978; Larew 1988). Leong et al. (2012) reported that treatments with petroleum oil were effective for preventing adult females of the Asiatic citrus psyllid, *Diaphorina citri* Kuwayama (Hemiptera: Psyllidae) from landing on citrus trees. The ovicidal and antiovideponent action of commercial products containing mineral oils could represent an interesting tool to reduce *G. funebrana* damage, as reported by Rizzo et al. (2018). A residual 2% v/v petroleum oil spray on wax-paper and apple-leaf substances significantly reduced the number of eggs laid by females of the obliquebanded leafroller, *Choristoneura rosaceana* (Harris) (Lepidoptera: Tortricidae) and caused over 99% egg mortality in some cases (Wins-Purdy et al. 2009).

Fungal diseases are also among the targets of petroleum oils. Field studies by Leong et al. (2012) in citrus orchards focused on the impact of treatments with 0.35% v/v petroleum oils have in the seasonal population changes, migration and dispersal of *D. citri*, the vector of the citrus disease huanglongbing (HLB). It was reported that the petroleum oils treatments reduce the number of diseased plants as compared to untreated control trees but also as compared to treated trees with triazophos, cypermethrin or chlorpyrifos.

Residues were found below the limit of quantification in apples (var. Red delicious) and in the soil under the canopy of the trees, based on a residue analysis that was conducted after one day of application with the recommended label dose of a commercial paraffinic oil during spring and summer season (Ahmad et al. 2018). According to Tan et al. (2005), the distribution of petroleum oils (nC24, UR > 99.8%) in citrus was mainly detected in the outer cortex of stems of the citrus seedlings after 10 days of application with 4% v/v oil. Furthermore, in navel oranges trees treated within a range of concentrations using an nC23 oil, deposits were detected after 10 months of applications in stems of sprayed flushes or new unsprayed flushes which were produced 4 to 5 months after the oil application (Tan et al. 2005). However, the authors did not report if the deposits were within the acceptable range of residues.

### Use in other crops and application scenarios

Since modern formulations of petroleum oils are considered as biorational insecticides, their application sites have been further expanded, including greenhouses, aquatic areas and animal premises, or even residential premises such as commercial industrial or medical establishments (US EPA 2007). Hence, petroleum oils efficacy has been evaluated in a broad range of host plants and pest species, including fruits (Liu and Stansly 1994; Northover and Schneider 1996; Liang and Liu 2002), vegetables (Reddy and Bautista 2012), flowers and ornamental plants (Miller 1989; Larew and Locke 1990; Marcinek et al. 2018), industrial and forage crops (Mensah et al. 1995, 2005; Kumar 2015), and pests species of health importance, such as mosquitoes (Micks 1970; Micks and Berlin 1970), bed bugs (Zha et al. 2018) and external parasites of livestock (USDA 2015).

#### Vegetables and fruits

Several researches have been conducted to evaluate the benefits of using petroleum oils as pesticides in fruits and vegetables. Kallianpur et al. (2002) examined the insecticidal efficacy of the petroleum oils in tomatoes, based on the mortality rates of the most common pests found in these crops, such as the tomato thrips, *Frankliniella schultzei* (Trybom) (Thysanoptera: Thripidae), the greenhouse whitefly, *Trialeurodes vaporariorum* (Westwood) (Hemiptera: Aleyrodidae), the tomato russet mite, *Aculops lycopersici* (Massee) (Acari: Eriophyidae), and the common brown leafhopper, *Orosius argentatus* (Evans) (Hemiptera: Cicadellidae). The work of Stansly and Conner (2005) also support the use of petroleum oils as a pest management tool for vegetable growers, since effective control was achieved against the silverleaf whitefly, *Bemisia argentifolii* Bellows and Perring (Hemiptera: Aleyrodidae), the broad mite, *Polyphagotarsonemus latus* Banks (Trombidiformes: Tarsonemidae), the green peach aphid, *Myzus persicae* (Sulzer) (Hemiptera: Aphididae), the southern armyworm, *Spodeptera eridania* Stoll (Noctuidae: Spodoptera), and the pepper weevil, *Anthonomus eugenii* Cano (Coleoptera: Curculionidae) in tomatoes and peppers. Herron et al. (1998) tested the mortality relationship of *M. persicae* between spray volume and oil concentration sprayed.

Liu et al. (2002) reported that there is a scope for using petroleum oils to reduce the total number of eggs and larvae of the Queensland fruit fly, *Bactrocera tryoni* (Froggatt) (Diptera: Tephritidae) in tomato fruits, based on the oviposition responses of the pest. The same authors indicated that the nC21 petroleum oils were found as the most effective among the other oils tested and suggested their use alone or in conjunction with baits to enhance the success especially in IPM programs. Nguyen et al. (2007) reported that petroleum oil-treated tomato fruits were about nine times less likely to be infested with *B. tryoni*. Xue et al. (2002) also stated that the oviposition by *F. schultzei* on French bean pods and tomato seedlings was significantly reduced due to deposits of a nC24 petroleum oil. However, the authors also reported phytotoxicity in the highest concentration tested (1.5 and 2%). Liu and Stansly (1994) found that petroleum oil as a dip proved to be at least as effective as the synthetic pyrethroid for *B. argentifolii* control on tomatoes under greenhouse and laboratory conditions.

It must be noted that *F. schultzei* and *O. argentatus* are one of the main vectors of tomato spotted wilt tospovirus and phytoplasmas associated with tomato big bud disease, respectively (Clift et al. 2002). In the same way, the control of the potato psyllid, *Bactericera cockerelli* (Sulc) (Hemiptera: Phyllidae) is of major importance in potatoes, tomatoes, and several other solanaceous crops, due to the potential of the species to transmit the bacterial pathogen *Candidatus* Liberibacter *solanacearum* (Alphaproteobacteria: Phyllobacteriaceae) to such crops (Lin et al. 2009). Therefore, the control or repellency provided by petroleum oils in these species, simultaneously prevents the further spread of the diseases, as reported by Clift et al. (2002) in field-grown fresh tomatoes and by Yang et al. (2010) in tomatoes under laboratory conditions. The authors also reported that the observed suppression of the diseases was equivalent to that obtained from using conventional pesticides. Other authors have also reported the efficacy of petroleum oils to suppress non-persistent viruses (Wang and Pirone 1996; Boquel et al. 2013; Khelifa 2017; Wróbel 2014; Galimberti and Alyokhin 2018).

#### Floriculture

Petroleum oils can be used also in floriculture. Marcinek et al. (2018) tested the influence of a prolong application of petroleum oils on bulb quality, quality of cut flowers and spread of viruses in tulip cultivations and reported that the oil preparations used at concentrations between 1.0 and 1.5% had no negative influence or phytotoxicity on stem and petals lengths at tulips grown in the field, or in the quality of obtained flowers during tulip forcing in a greenhouse. Furthermore, although the authors observed a positive effect in reducing diseases transferred by aphids, the tested oils could not ensure a complete plant protection from infections. No phytotoxicity was observed when four weekly petroleum spray of 1, 2, and 4% oil were applied on greenhouse-grown chrysanthemums by Larew and Locke (1990). Furthermore, the 2% (v/v) aqueous spray repelled adults for at least 11 days after spraying and it was toxic to newly hatched and third stage larvae of the whitefly, *Trialeurodes vaporariorum* (Westwood) (Hemiptera: Aleyrodidae) (Larew and Locke, 1990). Details about the use of petroleum oils as insecticides in amenity plants can be found in the work of Miller (1989).

#### Animal husbandry and public health

External applications of petroleum oils can be used for parasitic mite control in sheep, goats, cattle, hogs and other livestock, while these substances can also be used as lubricants. Petroleum oil products are also registered solely as mosquito larvicides/pupacides, acting as surface film agents. According to the US Center for Disease Control and Prevention (CDC), these products have important public health benefits, compared with the various other mosquito larvicides, because they are among the few currently available substances that are effective against pupae, able to provide a valuable option to an integrated mosquito control program. Nevertheless, petroleum oils are approved for use as direct, secondary direct and indirect food additives in human food and animal feed (USDA 2015; FDA 2021).

## Health and environmental concerns and legislation

### United States Environmental Protection Agency

An evidence-based risk assessment was published by US Environmental Protection Agency Office of Pesticide Programs (US EPA) in 2007, evaluating twelve different CAS numbers of petroleum-distilled oils, called in that work as aliphatic solvents which were diverted into mineral oils and aliphatic petroleum hydrocarbons (Table 1). According to the Agency, these oils had a low degree of acute toxicity and only slight eye irritation based on experiments conducted in rats and rabbits. Moreover, no subchronic and chronic toxicity reported, and hence, declaring the oils as virtually non-toxic by the oral or dermal route. Limited toxicity causing irritating effects via the inhalation route was reported, but this was stated as a result of the physical properties of oils rather than their chemical composition. The ecological toxicity assessment indicated no toxic effects of oils to mammals and birds, but direct application upon bird eggs may impairing their hatching. Contact toxicity testing suggested that the compounds are virtually safe to honey bees.

**Table 1:** Description of the various active ingredients found in mineral oil pesticides

Chemical name	CAS#	Description
Mineral oil, Oil mist (mineral)	8012–95-1	Liquid hydrocarbons obtained from petroleum
Mineral oil; Hydrocarbons oils; Paraffin liquid	8020–83-5	A mixture of liquid hydrocarbons obtained from petroleum
White mineral oil; Petroleum	8042–47-5	A highly refined petroleum mineral oil consisting of a complex combination of hydrocarbons obtained from the intensive treatment of a petroleum fraction with sulfuric acid and oleum, or by hydrogenation, or by a combination of hydrogenation and acid treatment. Additional washing and treating steps may be included in the processing operation. It consists of saturated hydrocarbons having carbon numbers predominantly in the range of C15 through C50
Distillates, petroleum, solvent-refined light paraffinic	64741–89-5	A complex combination of hydrocarbons obtained as the raffinate from a solvent extraction process. It consists predominantly of saturated hydrocarbons having carbon numbers predominantly in the range of C15 through C30 and produces a finished oil with a viscosity of less than 100 SUS at 100°F (19cSt at 40 °C)
Distillates, petroleum, hydrotreated heavy paraffinic	64742–54-7	A complex combination of hydrocarbons obtained by treating a petroleum fraction with hydrogen in the presence of a catalyst. It consists of hydrocarbons having carbon numbers predominantly in the range of C20 through C50 and produces a finished oil of at least 100 SUS at 100°F (19cSt at 40 °C). It contains a relatively large proportion of saturated hydrocarbons
Distillates, petroleum, hydrotreated light paraffinic	64742–55-8	A complex combination of hydrocarbons obtained by treating a petroleum fraction with hydrogen in the presence of a catalyst. It consists of hydrocarbons having carbon numbers predominantly in the range of C15 through C30 and produces a finished oil with a viscosity of less than 100 SUS at 100°F (19cSt at 40 °C). It contains a relatively large proportion of saturated hydrocarbons
Distillates, petroleum, solvent-dewaxed light paraffinic	64742–56-9	A complex combination of hydrocarbons obtained by removal of normal paraffins from a petroleum fraction by solvent crystallization. It consists predominantly of hydrocarbons having carbon numbers predominantly in the range of C15 through C30 and produces a finished oil with a viscosity of less than 100 SUS at 100°F (19cSt at 40 °C)
Distillates, petroleum, solvent-dewaxed heavy paraffinic	64742–65-0	A complex combination of hydrocarbons obtained by removal of normal paraffins from a petroleum fraction by solvent crystallization. It consists predominantly of hydrocarbons having carbon numbers predominantly in the range of C20 through C50 and produces a finished oil with a viscosity not less than 100 SUS at 100° F (19cSt at 40 °C)
Lubricating oils, petroleum C15-30, hydrotreated neutral oil based, containing. solvent deasphalted residual oil	72623–84-8	A complex combination of hydrocarbons obtained by treating light vacuum gas oil, heavy vacuum gas oil, and solvent deasphalted residual oil with hydrogen in the presence of a catalyst in a two-stage process with dewaxing being carried out between the two stages. It consists predominantly of hydrocarbons having carbon numbers predominantly in the range of C15 through C30 and produces a finished oil having a viscosity of approximately 10cSt at 40 °C (104°F). It contains a relatively large proportion of saturated hydrocarbons
Lubricating oils, petroleum, C15-30, hydrotreated neutral oil-based	72623–86-0	A complex combination of hydrocarbons obtained by treating light vacuum gas oil and heavy vacuum gas oil with hydrogen in the presence of a catalyst in a two-stage process and dewaxing being carried out between the two stages. It consists predominantly of hydrocarbons having carbon numbers predominantly in the range of C15 through C30 and produces a finished oil having a viscosity of approximately 15cSt at 40 °C. It contains a relatively large proportion of saturated hydrocarbons
Lubricating oils, petroleum, C20-50, hydrotreated neutral oil-based	72623–87-1	A complex combination of hydrocarbons obtained by treating light vacuum gas oil, heavy vacuum gas oil and solvent deasphalted residual oil with hydrogen in the presence of a catalyst in a two-stage process with dewaxing being carried out between the two stages. It consists primarily of hydrocarbons having carbon numbers predominantly in the range of C20 through C50 and produces a finished oil with a viscosity of approximately 32cSt at 40 °C. It contains a relatively large proportion of saturated hydrocarbons

Source: (US EPA 2007)

Regarding the toxicity evaluation towards the estuarine/marine and freshwater organisms, the results have shown virtually non-toxic effects. However, adverse effects on oyster shell deposition and in *Daphnia magna* Straus (Cladocera: Daphniidae) have been reported. The environmental fate assessment indicated that petroleum oils exhibit very poor migration and low potential for volatility due to their physical properties (low vapor pressures, very low solubility in water, high octanol–water partition coefficients, and high sorption to organic matter) (US EPA 2007). Overall, essentially no concerns have been indicated about the terrestrial effects of petroleum oils and most aquatic organisms, and the reported adverse effects may be probably due to the off-site spray drift of the compounds. More details about the current utilization of the commercial petroleum oils with respect to their CAS numbers can be found in the technical evaluation reports of the USDA (2015, 2019).

### European Food Safety Administration

The European Food Safety Administration (EFSA) Scientific Panel on Food Additives and Nutrient Sources added to Food (ANS) in 2009 published a scientific opinion evaluating the safety of petroleum oils as a food additive based on the results of Trimmer (2001) and Trimmer et al. (2004). The 2-year study in rats assessed the chronic toxicity and carcinogenicity of two mineral oils with specific viscosity values (CAS 8042–47-5) (Trimmer 2001; Trimmer et al. 2004). The results shown no adverse effects on survival, body weight, food consumption, clinical signs, clinical chemistry, haematology, and no treatment-related adverse changes were seen at necropsy or by microscopy. The Agent concluded that no carcinogenic effect was observed in rats and no safety concerns with respect to genotoxicity occurred (EFSA 2009). Moreover, no neurotoxic or teratogenic effect or adverse effect on fertility is either expected upon administration of the tested mineral oils (EFSA 2008; EFSA 2013).

In a peer review about the risk assessment of paraffin oils having hydrocarbons with chain lengths in the range of C_17_ to C_31_, EFSA (2008) summarized all the available data about the environmental fate of the substances. Regarding the biodiversity exposure to petroleum oils, EFSA (2008) evaluated the ecotoxicology of petroleum oils (CAS 8042–47-5). Studies conducted according to specific guidance documents about the risk assessment for birds, mammals and non-target arthropods (Mensah et al. 1995; Gunatilleka and Poole 1999; Stansly et al. 2002; Koster et al. 2019). Through these studies, the aliphatic hydrocarbons (paraffins) were considered as biochemically inert substances in both humans and animals. It was also stated that the paraffin oil (CAS 8042–47-5) is in accordance with the European Pharmacopeia, since such oils have for decades been used as a laxative intestinal lubricant without any harm on proper use to the patient (EFSA 2008).

Aquatic and terrestrial toxicology of the paraffinic oils was also thoroughly discussed by EFSA (2008). It was reported that proper use of paraffin oils expected to have low risk for bees and other arthropod species, earthworms and non-target plants. In addition, since the paraffin oils are a mixture of hydrocarbons with a simple structure, the substance is expected to biodegrade when in soil via oxidation and hydrocarbon chain splitting, with the final degradation product to be CO_2_. In addition, the persistence and degradation of the active substance of a commercial paraffinic oil (CAS 8042–57-5) and their metabolites in soil were also calculated. Information about the fate and behavior of commercial petroleum oil products were also provided via indoor microcosm and ready biodegradability studies. The derived data indicated that the substance dissipated rapidly, so that the accumulation at the water surface or in the water column from multiple applications was unlikely. Additional data about the risk assessment of the active substances of paraffin oils with different CAS numbers are available in EFSA Scientific Report (2008), in EFSA Reasoned Report (2012) and in EFSA Scientific Opinion (2012).

## Nomenclature of commercial products

The complexity and variability of petroleum oils regarding their chemical composition, refinement methods, target pests and recommended application periods led to various commercial affiliations over the years, addressing these factors. Therefore, petroleum oil formulations may be referred by many names, including petroleum distillates, petroleum-derived spray oils or PDSOs, petroleum spray oils or PSOs, hydrocarbon oils, lubricating oils, narrow-range oils, white mineral oils, aliphatic solvents, paraffin oils, mineral oils, horticultural oils, agricultural oils, supreme oil, superior oils, Volck oils, dormant oils, foliage or foliar oils, or summer oils. These names may have generic or more specific meanings and are not entirely synonymous with one another.

According to the US EPA (2007), nearly 341 petroleum oil mixtures with identical composition and/or level of purity have been registered as different compounds, i.e., active ingredients with different CAS (Codex Alimentarius Commission) numbers, thanks to their alternative refining pathways. The CAS number provide information on the uses, physical and chemical properties, toxicological effects, dietary assessment, and the environmental fate and eco-toxicity of the oil (US EPA 2007). Thus, the end-use products exhibit a specific range of properties and their label instructions are directly associated with their CAS number (Table 1).

Therefore, petroleum oils referred as petroleum distillates, lubricating oils, narrow-range oils, supreme oil, superior oils, or Volck oils imply the processing methods used in crude petroleum oil and thus, their chemical or physical characteristics. For example, mineral oils refer to oils derived by crude petroleum oil, and petroleum or mineral distillates refer mostly to oils derived after distillation of crude oils without any further refinement of treatment (Walters 2020). Volck oils are named after the inventor (Volck 1929), who observed that distillates with specific viscosity and gravity, or emulsified distillates sprayed in smaller droplets had increased insecticidal efficacy and were less harmful to the plants. Likewise, the above term referred to oils with clearly attainable specifications and were named by Dr. P.J. Chapman and co-workers in 1947 (Johnson 1980).

Narrow-range oils are identified based on the distillation range and paraffinic oils have a relatively high proportion of aliphatic structures. The latter-day term of supreme oils are highly refined oils that include many narrow-range oils but may be distilled at higher temperatures and over a wider range. Additionally, superior oils are a category of narrow-range oils with low phytotoxicity (Cranshaw and Baxendale 2005). White mineral oils referred to highly refined oils contain negligible quantities of phytotoxic contaminants and high levels of saturated hydrocarbons (high UR values) having carbon numbers predominantly in the range of nC15 through nC50. Due to their refinery, white mineral oils are colorless and odorless, and are considered as the most refined and most consistent of the petroleum oils, sold also as pharmaceutical- or food-grade substances (USDA 2019). On the other hand, a different nomenclature is used to distinct the petroleum oils based on their recommended application periods (dormant, foliage and summer oils), with respect to their phytotoxic effects. Dormant oils are less refined oils with lower UR values (< 92%) and higher viscosity and distillation range and are used on deciduous trees between leaf-fall and bloom against over-wintering stages of pests and pathogens. On the contrary foliage or foliar oils are applied to actively growing vegetation and summer oils are referred to products applied during warmer weather. These oils are more volatile with lower distillation values and higher UR values than that of dormant oils.

Either way, horticultural or agricultural oils referred to products used as plant protectants against pests. These terms include all the aforementioned types plus oils derived from plant extracts (essential oils). Consequently, the definition horticultural is generic in terms of chemical composition and refinery. To ensure the maximum insecticidal efficacy with the lowest phytotoxicity, petroleum oils should not be applied upon moisture-stressed plants or young foliage, in higher than the recommended label doses or in combinations with sulfur-based sprays, or when the temperature is above 37.8 °C or below 4.4 °C and the humidity is below 50% (Miller 1989).

## Conclusions

1. It is apparent that oils’ utilization was once limited to early-season or dormant sprays in the interest of avoiding oil injury to green plant tissue. However, various improvements in the refinement methods have enhanced the manufacture of oil formulations with high effectiveness and low phytotoxicity.
2. Current specifications for synthesis, chemical purification and refining reflect more modern uses where advanced oil products are manufactured for specific treatments which are environmentally suitable, toxicologically safe and fit neatly into the integrated pest management concept. It was therefore inevitable for petroleum oils to gain more recognition of their usefulness in and compatibility with modern pest management approaches.
3. The increased interest about the petroleum oils can be depicted through the first conference with title “Spray Oils Beyond 2000 – Sustainable Pest and Disease Management” held in Sydney, in 1999. The conference was the first attribution in petroleum oils, bringing different perspectives to research and development. Topics including the chemistry, refining and formulation of petroleum oils, modes of action against arthropods or arthropod mortality and plant diseases, phytotoxicity and physiological effects on plants, environmental and health issues, use in pest and disease management and many others, indicate the prosperous future of petroleum oils in agriculture and public health.

## Data Availability

Not applicable.

## References

[CR1] Abd-Rabou S, Badary H, Ahmed N (2012). Control measures of two soft scale insects (Hemiptera: Coccidae) infesting guava and mango trees in Egypt. J Basic Appl Zool.

[CR2] Agnello A, Beattie CGA, Watson DM, Stevens ML, Rae DJ, Spooner-Hart RN (2002). Petroleum-derived spray oils: chemistry, history, refining, and formulation. Spray oils beyond 2000: sustainable pest and disease management.

[CR3] Ahmad MM, Wani AA, Sofi M, Ara I (2018). Mineral oil residues in soil and apple under temperate conditions of Kashmir. India Environ Monit Assess.

[CR4] Amiri-Besheli B (2009) Toxicity evaluation of Tracer, Palizin, Sirinol, Runner and Tondexir with and without mineral oils on *Phylocnistis citrella* Stainton. African Journal of Biotechnology 8:3382–3386. http://www.academicjournals.org/AJB

[CR5] Baker JM (1970). The effects of oils on plants. Environ Pollut.

[CR6] Baxendale RW, Johnson WT (1988). Evaluation of summer oil spray on amenity plants. J Arboric.

[CR7] Beattie GAC, Lru M, Watson DM, Clift AD, Jiang L (1995). Evaluation of petroleum spray oils and polysaccharides for control of *Phyllocnistis citrella* Stainton (Lepidoptera: Gracillaridae). J Aust Entomol Soc.

[CR8] Beattie GAC, Smith D (1997) Integrated pest management: sustainable pest control for the future based on the past? In: Proceedings of the International Society of Citriculture, Southern Africa, pp 51–58

[CR9] Borgan CE, Ludwig S, Metz BE (2006) Using oils as pesticides. In: Agrilife Communications and Marketing, The Texas A&M University System, E-419. http://insects.tamu.edu/extension/publications/epubs/e-419.cfm

[CR10] Boquel S, Giguere MA, Clark C, Nanayakkara U, Zhang JH, Pelletier Y (2013). Effect of mineral oil on *Potato virus Y* acquisition by *Rhopalosiphum padi*. Entomol Exp Appl.

[CR11] Buteler M, Stadler T (2011) A review on the mode of action and current use of petroleum distilled spray oils. In: Stoytcheva M (ed) Pesticides in the modern world - Pesticides use and management, InTech, pp 119–136. http://www.intechopen.com/books/pesticides-in-the-modern-world-pesticides-use-and-management/a-reviewon-the-mode-of-action-and-current-use-of-petroleum-distilled-spray-oils

[CR12] Chueca P, Garcera C, Molto E, Jacas JA, Urbaneja A, Pina T (2010). Spray deposition and efficacy of four petroleum-derived oils used against *Tetranychus urticae* (Acari: Tetranychidae). J Econ Entomol.

[CR13] Clift AD, Singh P, Bowyer J, Rose H, Tesoriero L, Beattie GAC, Rajakulendran V (2002) Use of horticultural mineral oils to control tospovirus- and phytoplasma associated diseases of tomato (*Lycopersicon esculentum* Mill. [Solanaceae: Solanaceae]). In: Beattie GAC, Watson DM, Stevens ML, Rae DJ, Spooner-Hart RN (eds) Spray oils beyond 2000: sustainable pest and disease management, University of Western Sydney, Sydney, pp 562–567

[CR14] Cranshaw WS, Baxendale B (2005) Insect control: horticultural oils. In: Insect Series, Home and Garden Extension , Colorado State University, pp 569

[CR15] Damavandian MR (2016). Comparison of mineral oil spray with current synthetic pesticides to control important pests in citrus orchards and their side effects. Arthropods.

[CR16] Damavandian MR (2007). Laboratory and field evaluation of mineral oil spray for the control of citrus red mite, *Panonychus citri* (McGregor). Acta Agric Scandinavica Sect B-Soil Plant Sci.

[CR17] Damavandian MR, Moosavi SFK (2014). Comparison of mineral spray oil, Confidor, Dursban, and Abamectin used for the control of *Phyllocnistis citrella* (Lepidoptera: Gracillaridae), and an evaluation of the activity of this pest in citrus orchards in northern Iran. J Plant Protect Res.

[CR18] Davidson NA, Dibble JE, Flint ML, Marer PJ, Guye A (1991) Managing insects and mites with spray oils. University of California Special Publication 3347

[CR19] De Ong ER, Knight H, Chamberlin JC (1927). A preliminary study of petroleum oil as an insecticide for citrus trees. Hilgardia.

[CR20] Ebeling W, Rockstein M (1974). Permeability of insect cuticle. The physiology of insecta.

[CR21] European Food Safety Authority (2012). Reasoned opinion on the review of the existing maximum residue levels (MRLs) for paraffin oil (CAS 64742–54-7) according to Article 12 of Regulation (EC) No 396/2005. EFSA J.

[CR22] European Food Safety Authority Panel On Contaminants In The Food Chain (CONTAM) (2012) Scientific Opinion on mineral oil hydrocarbons in food. EFSA J 10:2704

[CR23] European Food Safety Authority Panel On Food Additives And Nutrient Sources Added To Food (ANS) (2013) Scientific opinion on the safety assessment of medium viscosity white mineral oils with a kinematic viscosity between 8.5 – 11 mm^2^/s at 100 °C for the proposed uses as a food additive. EFSA Journal 11:3073 10.2903/j.efsa.2013.3073 PMC13159817 42124958

[CR24] European Food Safety Authority Panel On Food Additives And Nutrient Sources Added To Food (ANS) (2009). Scientific Opinion on the use of high viscosity white mineral oils as a food additive. EFSA J.

[CR25] European Food Safety Authority (EFSA) (2008). Conclusion on pesticide peer review regarding the risk assessment of the active substance paraffin oils (CAS 64742–46-7, 72623–86-0 and 97862–82-3). EFSA Scientific Report.

[CR26] English LL (1928) Some properties of oil emulsion influencing insecticidal efficiency III. Illinois Natural History Survey Bulletin 17:235–259. https://doi.org/10.21900/j.inhs.v17.284

[CR27] Erler F (2004). Oviposition deterrency and deterrent stability of some oily substances against the pear psylla *Cacopsylla pyri*. Phytoparasitica.

[CR28] Fernandez DE, Beers EH, Brunner JF, Doerr MD, Dunley JE (2001). Mineral oil inhibition of white apple leafhopper (Homoptera: Cicadellidae) oviposition. J Entomol Sci.

[CR29] Fernandez DE, Beers EH, Brunner JF, Doerr MD, Dunley JE (2005). Effects of seasonal mineral oil applications on the pest and natural enemy complexes of apple. J Econ Entomol.

[CR30] Fernandez DE, Beers EH, Brunner JF, Doerr MD, Dunley JE (2006). Horticultural mineral oil applications for apple powdery mildew and codling moth, *Cydia pomonella* (L.). Crop Prot.

[CR31] Food And Drug Administration (FDA) (2021) Substances added to food (formerly EAFUS). US Food and Drug Administration. Retrieved from https://www.cfsanappsexternal.fda.gov/scripts/fdcc/?set=FoodSubstances&sort=Sortterm_ID&order=ASC&startrow=1&type=basic&search=mineral

[CR32] Fiori BJ, Smith EH, Chapman PJ (1963). Some factors influencing the ovicidal effectiveness of saturated petroleum oils and isoparaffins. J Econ Entomol.

[CR33] French JV, Hutchinson EM (1980). Citrus red mite: a potentially damaging pest of Texas citrus. J Rio Grande Valley Horticultural Soc.

[CR34] Galimberti A, Alyokhin A (2018). Lethal and sublethal effects of mineral oil on potato pests. J Econ Entomol.

[CR35] Gerolt P (1983). Insecticides – their route of entry, mechanism of transport and mode of action. Biol Rev Cambridge Philos Soc.

[CR36] Gurwitsh L (1927). The scientific principles of petroleum technology. Nature.

[CR37] Gray GP, De Ong ER (1926). California petroleum insecticides. Laboratory and field tests. Ind Eng Chem.

[CR38] Gruse WA (1928) Petroleum and its products: a chemical discussion of the properties, refining and utilization of petroleum. McGraw-Hill Book Co., New York, pp 377

[CR39] Gunatilleka AD, Poole CF (1999). Models for estimating the non-specific aquatic toxicity of organic compounds. Anal Commun.

[CR40] Hagg G (1969). General and inorganic chemistry.

[CR41] Hadley NF (1994). Water relations of terrestrial Arthropods.

[CR42] Helmy EI, Kwaiz FA, El-Sahn OMN (2012). The usage of mineral oils to control insects. Egyp Acad J Biol Sci A Entomol.

[CR43] Herron GA, Beattie GAC, Kallianpur A, Barchia I (1998). Influence of spray volume and oil concentration on the efficacy of petroleum spray oil against *Myzus persicae* (Sulzer) (Hemiptera: Aphididae). Aust J Entomol.

[CR44] Hurst GH (1925) Lubricating oils, fats, and greases: their origin, preparation, properties, uses and analysis, Scott Greenwood and Co., London, pp 410

[CR45] Johnson WT (1980). Spray oils as insecticides. J Arboric.

[CR46] Kallianpur AS, Herron GA, Beattie GAC, Barchia I (2002) Potter spray tower bioassays of two horticultural mineral oils against tomato thrips, tomato russet mite and greenhouse whitefly adults, and common brown leafhopper nymphs. In: Beattie GAC, Watson DM, Stevens ML, Rae DJ, Spooner-Hart RN (eds) Spray oils beyond 2000: sustainable pest and disease management, University of Western Sydney, Sydney, pp 142–146

[CR47] Khelifa M (2017). Possible induction of potato plant defenses against Potato virus Y by mineral oil application. Eur J Plant Pathol.

[CR48] Kim D, Seo YD, Choi KS (2010). The effects of petroleum oil and lime sulfur on the mortality of *Unaspis yanonensis* and *Aculops pelekassi* in the laboratory. J Asia-Pacific Entomol.

[CR49] Koster S, Varela J, Stadler RH, Moulin J, Cruz-Hernandez C, Hielscher J, Lesueur C, Roïz J, Simian H (2019) Mineral oil hydrocarbons in foods: is the data reliable? Food Addit Contam: Part A 1-15. 10.1080/19440049.2019.1678770 10.1080/19440049.2019.1678770 31639315

[CR50] Kuhlmann B, Jacques DF (2002) Classifications, standards and nomenclature⎯mineral oils, agricultural mineral oils and horticultural mineral oils. In: Beattie GAC, Watson DM, Stevens ML, Rae DJ, Spooner-Hart RN (eds), Spray Oils Beyond 2000: Sustainable Pest and Disease Management. University of Western Sydney. pp. 29–38

[CR51] Kumar S (2015). Evaluation of petroleum spray oils against turnip aphid, *Lipaphis erysimi* (Kaltenbach) infesting oilseed brassica. Int J Bio-Resour Stress Manag.

[CR52] Larew HG (1988). Effects of 4 horticultural oils on whitefly oviposition. Insecticide Acaricide Tests.

[CR53] Larew HG, Locke JC (1990). Repellency and toxicity of a horticultural oil against whiteflies on chrysanthemum. HortScience.

[CR54] Leong SCTL, Abang F, Beattie A, Kueh RJH, Wong SK (2012). Impacts of horticultural mineral oils and two insecticide practices on population fluctuation of *Diaphorina citri* and spread of Huanglongbing in a citrus orchard in Sarawak. Sci World J.

[CR55] Liang G, Liu T (2002). Repellency of a kaolin particle film, surround, and a mineral oil, sunspray oil, to silverleaf whitefly (Homoptera: Aleyrodidae) on melon in the laboratory. J Econ Entomol.

[CR56] Liang W, Meats A, Beattie GAC, Spooner-Hart R, Jiang L (2010). Conservation of natural enemy fauna in citrus canopies by horticultural mineral oil: Comparison with effects of carbaryl and methidathion treatments for control of armored scales. Insect Sci.

[CR57] Lin H, Doddapaneni H, Munyaneza JE, Civerolo EL, Sengoda VG, Buchman JL, Stenger DC (2009). Molecular characterization and phylogenetic analysis of 16S rRNA from a new "Candidatus Liberibacter" strain associated with Zebra Chip disease of potato (*Solanum tuberosum* L.) and the potato psyllid (*Bactericera cockerelli* Sulc). J Plant Pathol.

[CR58] Liu ZM, Beattie GAC, Johnson D, Spooner-Hart R (2002) Influence of deposits of a horticultural mineral oil and selected fractions of paraffinic and naphthenic petroleum-derived oils on oviposition by Queensland fruit fly on tomato fruit. In: Beattie GAC, Watson DM, Stevens ML, Rae DJ, Spooner-Hart RN (eds) Spray oils beyond 2000: sustainable pest and disease management, University of Western Sydney, Sydney, pp 142–146

[CR59] Liu T, Stansly PA (1994). Toxicity and repellency of some biorational insecticides to *Bemisia argentifolii* on tomato plants. Entomol Exp Appl.

[CR60] Liu ZM, Beattie GAC, Hodgkinson M, Rose HA, Jiang L (2001). Influence of petroleum-derived spray oil aromaticity, equivalent n-paraffin carbon number and emulsifier concentration on oviposition by citrus leafminer, *Phyllocnistis citrella* Stainton (Lepidoptera: Gracillariidae). Aust J Entomol.

[CR61] Lockey KH (1988). Lipids of the insect cuticle: origin, composition and function. Comp Biochem Physiol Part B: Comp Biochem.

[CR62] Marcinek B, Karczmarz K, Szmagara M, Durlak W, Pogroszewska E (2018). Influence of a prolong application of mineral oils on bulb yields, quality of cut flowers and spread of viruses in tulip cultivation. Acta Scientiarum Polonorum Hortorum Cultus.

[CR63] Mazzella N, Molinet J, Syakti AD, Barriol A, Dodi A, Bertrand JC, Doumenq P (2005). Effects of pure n-alkanes and crude oil on bacterial phospholipids classes and molecular species determined by electrospray ionization mass spectrometry. J Chromatogr B.

[CR64] Mensah RK, Harris WE, Beattie GAC (1995). Response of *Helicoverpa* spp. (Lep: Noctuidae) and their natural enemies to petroleum spray oil in cotton in Australia. Entomophaga.

[CR65] Mensah R, Liang W, Coates R (2004) Petroleum spray oils- lubricating the path to IPM: Part I. Use of petroleum spray oil as insecticide to control *Helicoverpa* spp on commercial cotton fields. In: Proceedings of 2004 Australian Cotton Conference, Queensland, Australia, pp 675–681

[CR66] Mensah R, Liang W, Gibbs D, Coates R, Johnson D (2005). Evaluation of nC24 petroleum spray oil for activity against *Helicoverpa* spp. on commercial cotton fields in Australia. Int J Pest Manag.

[CR67] Mensah R, Liang W, Singleton A (2002) Improving the efficacy of nuclear polyhedrosis virus (NPV) and Bacillus thuringiensis (Bt) against *Helicoverpa* spp. on cotton with petroleum spray oil. In: Proceedings of the 11^th^ Australian Cotton Conference, Brisbane, Australia, pp 279–288

[CR68] Mensah R, Liang W, Singleton A (2001). Petroleum spray oils: key component of sustainable IPM in cotton. Proceedings 32^nd^ Scientific Conference of Australian Entomological Society.

[CR69] Micks DW (1970). Mosquito control agents derived from petroleum hydrocarbons IV. Further larval abnormalities produced by FLIT® MLO. J Econ Entomol.

[CR70] Micks DW, Berlin JA (1970). Continued susceptibility of *Culex pipiens quinquefasciatus* to petroleum hydrocarbons. J Econ Entomol.

[CR71] Miller FD (1989). The use of horticultural oils and insecticidal soaps for control of insect pests of amenity plants. J Arboric.

[CR72] Moore W, Graham SA (1918). A study of the toxicity of kerosene. J Econ Entomol.

[CR73] Najar-Rodríguez AJ, Lavidis NA, Mensah RK, Choy PT, Walter GH (2008). The toxicological effects of petroleum spray oils on insects – evidence for an alternative mode of action and possible new control options. Food Chem Toxicol.

[CR74] Najar-Rodríguez AJ, Walter GH, Mensah RK (2007). The efficacy of a petroleum spray oil against *Aphis gossypii* Glover on cotton. Part 1: Mortality rates and sources of variation. Pest Manag Sci.

[CR75] Neumann H, Rahimian I (1984). Petroleum Refining. Vol. 7. Geol Pet Geol Magazine.

[CR76] Nguyen VL, Meats A, Beattie GAC, Spooner-Hart R, Liu ZM, Jiang L (2007). Behavioural responses of female Queensland fruit fly, *Bactrocera tryoni*, to mineral oil deposits. Entomol Exp Appl.

[CR77] Nicetic O, Cho YR, Rae DJ (2011). Impact of physical characteristics of some mineral and plant oils on efficacy against selected pests. J Appl Entomol.

[CR78] Northover J, Schneider KE (1996). Physical modes of action of petroleum and plant oils on powdery and downy mildews of grapevines. Plant Dis.

[CR79] National Organic Standards Board (NOSB) (1995) National Organic Standards Board Meeting Minutes, Orlando, Florida, April 24–28, 1995

[CR80] Pearce GW, Chapman PJ (1952). Insecticidal efficacy of petroleum fractions and synthetic isoparaffins. Adv Chem.

[CR81] Rae DJ, Beattie GAC, Watson DM, Liu ZM, Jiang L (1996). Effects of petroleum spray oils without and with copper fungicides on the control of citrus leafminer, *Phyllocnistis citrella* Stainton (Lepidoptera: Gracillariidae). Aust J Entomol.

[CR82] Rae DJ, Watson DM, Huang MD, Cen YJ, Wang BZ, Beattie GAC, Liang WG, Tan BL, Liu DG (2000). Efficacy and phytotoxicity of multiple petroleum oil sprays on sweet orange (*Citrus sinensis* (L.)) and pummelo (*C. grandis* (L.)) in Southern China. Int J Pest Manag.

[CR83] Reddy GVP, Bautista JR (2012). Integration of the predatory mite *Neoseiulus californicus* with petroleum spray oil treatments for control of *Tetranychus marianae* on eggplant. Biocontrol Sci Tech.

[CR84] Riedl H, Halaj J, Kreowski WB, Hilton RJ, Westigard PH (1995). Laboratory evaluation of mineral oils for control of codling moth (Lepidoptera: Tortricidae). J Econ Entomol.

[CR85] Riley CV (1891). The outlook for applied entomology. Insect Life.

[CR86] Riley CV (1892). The kerosene emulsion: its origin, nature, and increasing usefulness. Soc Promotion Agr Sci Proc Ann Meetings.

[CR87] Rizzo R, Caleca V, Lombardo A, Lo Verde G (2018). Effectiveness of spinosad and mineral oil based commercial products on oviposition and egg hatching of *Grapholita funebrana* Treitschke. REDIA.

[CR88] Rizzo R, Lo Verde G, Lombardo A (2012). Effectiveness of spinosad and mineral oil for control of *Grapholita funebrana* Treitschke in organic plum orchards. Supplemento New Medit.

[CR89] Rohrbaugh PW (1941). Physiological effects of petroleum oil sprays on citrus. J Econ Entomol.

[CR90] Rourke BC, Gibbs AG (1999). Effects of lipid phase transitions on cuticular permeability: model membrane and in situ studies. J Exp Biol.

[CR91] Rowlinson JS, Freeman PI (1961). Lower critical solution points in hydrocarbon mixtures. Pure Appl Chem.

[CR92] Sazo L, Araya JE, Esparza S (2008). Control of San Jose scale nymphs, *Diaspidiotus perniciosus* (Comstock), on almond and apple orchards with pyriproxyfen, phenoxycarb, chlorpyrifos and mineral oil. Chilean J Agric Res.

[CR93] Sepasi M, Damavandian MR, Besheli BA (2018). Mineral oil barrier is an effective alternative for suppression of damage by white snails. Acta Agric Scandinavica.

[CR94] Shepard HH (1939). Chemistry and toxicity of insecticides.

[CR95] Smith EH (1952). Tree spray oils. Agricultural Applications of Petroleum Products.

[CR96] Stadler T, Buteler M (2009). Modes of entry of petroleum distilled spray-oils into insects: a review. Bull Insectol.

[CR97] Stadler T, Schang MM, Zerba E (1996). Caracterización fisicoquímica y toxicológica de algunos aceites minerales de uso fitosanitario. Revista De Investigaciones Agropecuarias.

[CR98] Stansly PA, Conner JM (2005). Crop and insect response to horticultural mineral oil on tomato and pepper. Proc Flo State Hort Soc.

[CR99] Stansly PA, Liu T-X, Schuster DJ (2002) Effects of horticultural mineral oils on a polyphagous whitefly, its plant hosts and its natural enemies. In: Beattie GAC, Watson DM, Stevens ML, Rae DJ, Spooner-Hart RN (eds) Spray oils beyond 2000: sustainable pest and disease management, University of Western Sydney, Sydney, pp 120–133

[CR100] Sugiura M, Horibe Y, Kawada H, Takagi M (2008). Insect spiracle as the main penetration route of pyrethroids. Pestic Biochem Physiol.

[CR101] Swingle HS, Snapp OI (1931). Petroleum oils and oil emulsions as insecticides, and their use against the San Jose scale on peach trees in the south. USDA Technical Bulletin.

[CR102] Tan BL, Reddy N, Sarafis V, Beattie GAC, Spooner-Hart R (2005). Noninvasive localization of petroleum-derived spray oil in plants with chemical shift selective magnetic resonance imaging. HortScience.

[CR103] Taverner P (2002) Drowning or just waving? A perspective on the ways petroleum-based oils kill arthropod pests of plants. In: Beattie GAC, Watson DM, Stevens ML, Rae DJ, Spooner-Hart RN (eds) Spray oils beyond 2000: sustainable pest and disease management, University of Western Sydney, Sydney, pp 78–87

[CR104] Taverner PD, Gunning RV, Kolesik P, Bailey PT, Inceoglu AB, Hammock B, Roush RT (2001). Evidence for direct neural toxicity of a “light” oil on the peripheral nerves of lightbrown apple moth. Pestic Biochem Physiol.

[CR105] Taverner PD, Sutton C, Cunningham N, Dyson C, Lucas N, Myers SW (2011). Efficacy of several insecticides alone and with horticultural mineral oils on light brown apple moth (Lepidoptera: Tortricidae) eggs. J Econ Entomol.

[CR106] Trammel K (1965) Properties of petroleum oils in relation to performance as citrus tree sprays in Florida. Dissertation, University of Florida Citrus Experimental Station, USA

[CR107] Trimmer GW (2001) Final report project number: 105970, test substance white oil (mrd-97–059), combined chronic toxicity/carcinogenicity study of white oil in fischer rats, and appendix BW. ExxonMobil Biomedical Sciences, Inc., New Jersey, USA

[CR108] Trimmer GW, Freeman JJ, Priston RAJ, Urbanus J (2004). Results of dietary chronic toxicity studies of high viscosity (P70 and P100) white mineral oils in Fischer rats. Toxicol Pathol.

[CR109] U.S. Department Of Agriculture (USDA) (2015) Mineral oil-livestock. Technical evaluation report compiled by pesticide research institute for the USDA national organic program

[CR110] U.S. Department Of Agriculture (USDA) (2019) Narrow-range oils – crops. Technical evaluation report compiled by nexight group for the USDA national organic program

[CR111] U.S. Environmental Protection Agency (US EPA) (2007) Revised reregistration eligibility decision (red) document for the aliphatic solvents case 1050 (mineral oil and aliphatic petroleum hydrocarbons). US Environmental Protection Agency

[CR112] Volck WH (1929) Insecticide and process of making and using the same to protect plants. US Patent Office 1,707,465

[CR113] Volck WH (1903). Spraying with distillates. Calif Agric Experiment Station Bull.

[CR114] Walters C (2020) Petroleum. In: (ed) Kirk-Othmer Encyclopedia of Chemical Technology, John Wiley & Sons Inc., 10.1002/0471238961.1518090702011811.a01.pub3

[CR115] Wang RY, Pirone TP (1996). Mineral oil interferes with retention of tobacco etch potyvirus in the stylets of *Myzus persicae*. Phytopathology.

[CR116] Wedding BT, Riehl LA, Rhoads WA (1952). Effect of petroleum oil spray on photosynthesis and respiration in citrus leaves. Plant Physiol.

[CR117] Weissling TJ, Lewis TM, Mcdonough LM, Horton DR (1997). Reduction in pear psylla (Homoptera: Psyllidae) oviposition and feeding by foliar application of various materials. Can Entomol.

[CR118] Wigglesworth V (1941). Oils aiding loss of water from the cuticle. Nature.

[CR119] Willett M, Westigard PH (1988). Using horticultural spray oils to control orchard pests. Pacific Northwest Insect Control Handbook.

[CR120] Wins-Purdy AH, Whitehouse C, Judd GJR, Evenden ML (2009). Effect of horticultural oil on oviposition behaviour and egg survival in the obliquebanded leafroller (Lepidoptera: Tortricidae). Can Entomol.

[CR121] Wróbel S (2014). Efficacy of mineral oil-insecticide mixtures for protection of potato tubers against PVY and PVM. Am J Potato Res.

[CR122] Xue YG, Watson DM, Nicetic O, Beattie GAC (2002). Impact of nC24 horticultural mineral oil deposits on the behaviour of *Frankliniella schultzei* (Trybom) (Thysanoptera: Thripidae). General Appl Entomol.

[CR123] Yamamoto A, Yoneda H, Hatano R, Asada M (1995). Genetic analysis of hexythizox resistance in the citrus red mite, *Panonychus citri* McGregor. J Pestic Sci.

[CR124] Yamamoto A, Yoneda H, Hatano R, Asada M (1996). Realized heritability estimates of hexythiazox resistance in the citrus red mite, *Panonychus citri* McGregor. J Pestic Sci.

[CR125] Yang X-B, Zhang Y-M, Hua L, Peng L-N, Munyaneza JE, Trumble JT, Liu T-X (2010). Repellency of selected biorational insecticides to potato psyllid, *Bactericera cockerelli* (Hemiptera: Psyllidae). Crop Prot.

[CR126] Zha C, Wang C, Li A (2018). Toxicities of selected essential oils, silicone oils, and paraffin oil against the common bed bug (Hemiptera: Cimicidae). J Econ Entomol.

[CR127] Zwick RW, Westigard PH (1978). Prebloom petroleum oil applications for delaying pear psylla (Homoptera: Psyllidae) oviposition. Can Entomol.

